# Large-Scale Purification of r28M: A Bispecific scFv Antibody Targeting Human Melanoma Produced in Transgenic Cattle

**DOI:** 10.1371/journal.pone.0140471

**Published:** 2015-10-15

**Authors:** Katrin Spiesberger, Florian Paulfranz, Anton Egger, Judith Reiser, Claus Vogl, Judith Rudolf-Scholik, Corina Mayrhofer, Ludger Grosse-Hovest, Gottfried Brem

**Affiliations:** 1 Department of Biomedical Sciences, Institute of Animal Breeding and Genetics, University of Veterinary Medicine Vienna, Vienna, Austria; 2 Christian Doppler Laboratory for Innovative Immunotherapy, University of Veterinary Medicine Vienna, Vienna, Austria; 3 Department of Agrobiotechnology (IFA Tulln), Institute of Biotechnology in Animal Production, University of Natural Resources and Applied Life Sciences Vienna, Tulln, Austria; 4 Department of Immunology, Institute for Cell Biology, University of Tübingen, Tübingen, Germany; Emory University School of Medicine, UNITED STATES

## Abstract

**Background:**

30 years ago, the potential of bispecific antibodies to engage cytotoxic T cells for the lysis of cancer cells was discovered. Today a variety of bispecific antibodies against diverse cell surface structures have been developed, the majority of them produced in mammalian cell culture systems. Beside the r28M, described here, no such bispecific antibody is known to be expressed by transgenic livestock, although various biologicals for medical needs are already harvested—mostly from the milk—of these transgenics. In this study we investigated the large-scale purification and biological activity of the bispecific antibody r28M, expressed in the blood of transgenic cattle. This tandem single-chain variable fragment antibody is designed to target human CD28 and the melanoma/glioblastoma-associated cell surface chondroitin sulfate proteoglycan 4 (CSPG4).

**Results:**

With the described optimized purification protocol an average yield of 30 mg enriched r28M fraction out of 2 liters bovine plasma could be obtained. Separation of this enriched fraction by size exclusion chromatography into monomers, dimers and aggregates and further testing regarding the biological activity revealed the monomer fraction as being the most appropriate one to continue working with. The detailed characterization of the antibody’s activity confirmed its high specificity to induce the killing of CSPG4 positive cells. In addition, first insights into tumor cell death pathways mediated by r28M-activated peripheral blood mononuclear cells were gained. In consideration of possible applications *in vivo* we also tested the effect of the addition of different excipients to r28M.

**Conclusion:**

Summing up, we managed to purify monomeric r28M from bovine plasma in a large-scale preparation and could prove that its biological activity is unaffected and still highly specific and thus, might be applicable for the treatment of melanoma.

## Introduction

30 years ago, Staerz and colleagues discovered the potential of bispecific antibodies to engage cytotoxic T cells for the lysis of cancer cells [1]. Since then, a plethora of recombinant bispecific antibody formats has been developed for therapeutic applications [2]. Recently, antibodies derived from single-chain variable antibody fragments (scFv), have been in the focus of research, e.g. tandem scFv molecules, diabodies, single-chain diabodies, tandem single-chain diabodies and various derivates thereof [2–8].

So far, most bispecific antibodies that mediate the killing of cancer cells harbor a CD3 binding site for the efficient activation of T cells [4, 5, 7, 9]. Another target site is CD28. As already discovered in the late 80ies the anti-CD28 monoclonal antibody 9.3 provides a signal bypassing accessory cell requirement in T cell activation [10]. Since then, many bispecific antibodies harboring a CD28 binding site have been described, that are capable of activating T cells without additional TCR/CD3 engagement [11–15]. This effect was explained by the formation of a synaptic cleft between the T cell and the engaged cancer cell, generated by the close proximity of these cells. This enables the T cell to release its toxins into that cleft, resulting in a far higher local concentration of toxins in the cleft than by undirected release [16].

Since the detrimental outcome of a clinical study from 2006 in which the application of a superagonist anti-CD28 monoclonal antibody (TGN1412) caused severe inflammatory responses [17], heightened awareness is still paid to antibodies harboring CD28 binding sites. Remarkable, as recently published, the same antibody, now under the name TA08, successfully completed phase I testing [18].

In this study, the characteristics of the bispecific antibody r28M, a tandem scFv antibody expressed in the blood of transgenic cattle are revisited. To generate these animals, primary fetal fibroblasts were transfected with the r28M construct, selected *in vitro* and used for nuclear transfer as described by Grosse-Hovest et al. [19]. The r28M construct consists of the 9.3 anti-CD28 scFv fragment and the 9.2.27 anti-CSPG4 scFv fragment, joined by a 19 amino acid peptide linker, and an additional c-myc-tag [19]. Noteworthy, in contrast to the above mentioned TGN1412, the r28M antibody has no Fc-portion.

CSPG4 is expressed on cancer cells as well as angiogenic vasculature and is associated with an aggressive disease course in several malignancies including melanoma and glioblastoma [20, 21]. Although melanoma accounts for less than 2% of all skin cancer cases, it accounts for the vast majority of skin cancer deaths, which is predominantly due to the spread of metastases [22]. Here the application of r28M could offer a new therapeutic field. Highly target-specific scFv antibodies like the r28M, distributed via the blood stream, are able to penetrate tumors rapidly, evenly and persist longer than for example Fab-antibodies [23].

Hence, the characterization of the mode of action of bispecific antibodies is central to this field of research, so is the optimal production of these antibodies. The majority of commercially manufactured antibodies originates from mammalian cell culture systems [24]. In spite of the progress during the last decade, there are still issues to solve, including the massive costs of building production plants and the up-scaling of the production volume in bioreactors [25]. Other challenges are the control of product quality and relatively low production levels as well as carbon dioxide concentrations, the minimization of possible contaminations [24] and the stability and reliability of glycosylation patterns and other post-translational modifications [26]. To overcome these problems current approaches that use transgenic livestock for the production of recombinant pharmaceuticals are pursued. [27]. These active agents are usually collected from milk or blood. Several products purified from milk have already been tested in clinical trials, as reviewed by Kues and Niemann [28]. In 2006, the first pharmaceutical product from a transgenic farm animal, antithrombin III (ATryn from GTC-Biotherapeutics, USA) which is expressed in the mammary gland of transgenic goats, has been approved by the European Medicines Agency [28]. Other proteins like fibrinogen or albumin are also produced in the milk of transgenic goats and rabbits, but up to now, except for r28M, no scFv antibody has been designed to be expressed by cattle, neither in their milk nor in their blood [26].

In the present study, we optimized the purification process of r28M out of blood samples from transgenic cattle for large-scale purposes. Furthermore, we provide new insights into the biological activity of the purified antibody in detail *in vitro*.

## Materials and Methods

### Ethics statement

Human buffy coat preparations, by-products of plasmapheresis, from healthy male donors between 25 and 45 years of age, were purchased from the local transfusion service (Red Cross Blood Service; Vienna, Austria).

The project “plasmapheresis and gaining of bovine serum” was discussed by the local ethics committee and in accordance with the German Welfare Act (2006; BGBl.I S.1206, 1313; BGBl. I S. 1308) approved by the government of Upper Bavaria (registration date: 14.12.2009; file number: 55.21-1-54-2531.6-15-09).

### Cell culture

A-375 (#300110) and U-251 MG (#300385) cell lines were purchased from CLS-cell line service (Eppelheim, Germany). IPC-298 (#ACC 251) cells were purchased from DSMZ-Deutsche Sammlung von Mikroorganismen und Zellkulturen; Braunschweig, Germany. Except for U-251 MG, all cell lines were positive for CSPG4. The cell lines were grown at 37°C in a humidified atmosphere containing 5% CO_2_ in media recommended by the manufacturer. All cell culture media and reagents were purchased from GE Healthcare (Buckinghamshire, GB).

### Isolation of cells

Peripheral blood mononuclear cells (PBMC) were isolated from human buffy coat preparations by density gradient centrifugation using the lymphocyte separation medium LSM-1077 and the blood separation tubes (GE Healthcare) according to the manufacturer’s protocol.

CD4^+^, CD8^+^ and CD56^+^ cells were purified from PBMC using the MACS® CD4, CD8 and CD56 MicroBeads. The positive selection was performed according to the manufacturer’s instructions (Miltenyi Biotech; Bergisch Gladbach, Germany).

After purification and/or separation the cells were suspended in freezing medium (50% fetal calf serum, 40% full medium, 10% dimethyl sulfoxide) and stored at -152°C. PBMC and subpopulations were thawed 24 hours prior to experimental use and cultured in RPMI-1640 supplemented with heat-inactivated 10% fetal calf serum (FCS) (GE Healthcare) as described above.

### Animals and plasma collection

Ten cattle of the breed Fleckvieh, aged 19–53 months, were used as donors. All animals were clinically healthy and regularly examined by a veterinarian before and after plasmapheresis (“plasmapheresis and gaining of bovine serum” approved by the government of Upper Bavaria; registration date: 14.12.2009; file number: 55.21-1-54-2531.6-15-09). All efforts were made to minimize suffering.

Plasmapheresis was performed with an automated plasmapheresis machine (Octo Nova®, DIAMED Medizintechnik GmbH; Cologne, Germany). Under aseptical conditions, a 12 gauge double lumen catheter (Arrow®, Teleflex Medical; Athlone, Ireland) was placed into the jugular vein of the cattle and the animal was connected to the plasmapheresis machine. R28M containing whole blood was spiked with acid citrate dextrose, pH 5, at a ratio of 1:20. Following whole blood collection, the separated cellular blood components were continuously reinfused, the harvested plasma was replaced by an equal amount of 0.9% saline.

Plasma samples were stored at -20°C until they were used for further purification steps.

### Purification and fractionation of the r28M protein

For purification of r28M, two liters of plasma were thawed at 4°C and centrifuged for 15 min at 6000 x g. Proteins in the supernatant were precipitated by adding a 35% polyethylene glycol 400 (PEG) solution or a 50% saturated ammonium sulfate (AS) solution. Following one hour of incubation at 4°C, the solution was centrifuged for 20 min at 6000 x g. The precipitated proteins were resuspended in 500 ml phosphate buffered saline (PBS) and loaded on a HiTrap^TM^ Protein L 5 ml sepharose column (GE Healthcare). Bound proteins were eluted stepwise with 50 mM glycine, 50 mM citric acid and 0.1 M NaCl (pH 3.5) followed by 50 mM glycine and 50 mM citric acid (pH 2).

After neutralization with 0.5 M disodium hydrogen phosphate (pH 8.8) the buffer was exchanged to 20 mM disodium hydrogen phosphate and 150 mM NaCl (pH 7.2) and the fraction was passed over a HiTrap^TM^ Protein A HP 1 ml column or over a HiTrap^TM^ Protein G HP 1 ml column (both GE Healthcare). Size exclusion chromatography (SEC) was performed via HiLoad^TM^ 16/60 Superdex^TM^ 200 pg (GE healthcare) using PBS as solvent buffer. The protein concentrations of the individual fractions were determined by a bichinconinic acid (BCA) Protein Assay Kit (Thermo Scientific; Rockford, USA) according to the manufacturer‘s protocol. All samples were stored at -80°C.

The enriched bispecific antibody fraction was further charged with different detergents, namely Tween® 20, Tween® 80, sodium-deoxycholate (Na-deoxycholate) or saponin diluted in PBS as indicated in the results section.

### Characterization of r28M properties

#### Measurement of viable tumor cells

Viable tumor cells were determined by a functional assay as described previously [11] with some minor modifications. Briefly, tumor cells were incubated in quadruplets in 96-well plates (1 x 10^4^/well) together with PBMC (target-effector ratio = 1:10) and purified r28M as indicated in a final volume of 150μl. After 3 days the PBMC were removed by multiple washing steps with PBS and water-soluble tetrazolium salt (WST) (Roche Diagnostics; Mannheim, Germany) was added. The amount of remaining viable adherent tumor cells was determined by measuring the optical density at 450 nm using an ELISA reader (Epoch Microplate Spectrophotometer, Biotek; Bad Friedrichshall, Germany). The percentage of viable tumor cells was calculated as follows: A_sample_/A_max_, with A_max_ being the optical density generated by tumor cells that have been incubated with PBMC but without r28M. Background subtraction was performed using WST dye only.

#### FACS analysis

For the analysis of PBMC populations, cells were incubated with following antibodies according to the manufacturer’s instructions: monoclonal mouse anti-human-CD3-FITC (cat.no. 11–0039, clone HIT3a), monoclonal mouse anti-human-CD4-PerCP/Cy5.5 (cat.no. 45–0048, clone OKT4), monoclonal mouse anti-human-CD8-PE (cat.no. 12–0086, clone OKT8) and monoclonal mouse anti-human-CD56-APC (cat.no. 17–0567, clone CMSSB) (eBioscience; Vienna, Austria). The viability of cells was determined by incubation with propidium iodide (ATT Bioquest; Sunnyvale, USA). Cells were analyzed using a FACSCanto II (BD; San Jose, USA) equipped with FACSDiva Software (BD).

#### Protein analysis

SDS-polyacrylamide gel electrophoresis (SDS-PAGE) was performed using 8% polyacrylamide (PAA) gels and proteins were either visualized by silver staining or transferred onto nitrocellulose membranes (Protran, Whatman; Kent, UK). For visualizing proteins by silver staining the PAA gel was fixed in 50% methanol with 5% acetic acid. After several washing steps the gel was sensitized in 0.02% sodium thiosulfate and washed again before incubated in cold 0.2% silver nitrate with 0.02% formaldehyde. The gel was washed and developed in 0.05% formaldehyde with 3% sodium carbonate. The staining was determined by incubation in 5% acetic acid. The band densities were analyzed using Quantity One Software (Version 4.6.9; Biorad; Hercules, USA).

For the detection of r28M and BSA, anti-c-myc and anti-BSA Western Blots were performed. Membranes were blocked with 0.5% polyvinyl alcohol in PBS containing 0.05% Tween® 20 overnight at 4°C. Subsequently they were incubated with polyclonal sheep anti-BSA-HRP (1:2500) (cat.no. SAL-10P, ICL Inc.; Portland, USA) or monoclonal mouse anti-c-myc-HRP (1:3000) (cat.no. R951-25, Invitrogen/Life Technologies; Carlsbad, USA) in PBS containing 0.05% Tween® 20. After that the membranes were rinsed for one minute in 0.01 M Tris-HCl (pH 6) and developed via a tetramethylbenzidine (TMB) solution (0.24% TMB and 0.8% dioctylsulfosuccinate sodium salt solved in ethanol, diluted 1:4 in 0.15 M citric acid, pH 5, and 5 μl 37% H_2_O_2_ per 10 ml solution were added). The color reaction was stopped by rinsing the membranes in ddH_2_O.

#### Detection of caspase subunits

For the detection of caspase subunits, 8 x 10^5^ tumor cells were incubated together with 8 x 10^6^ PBMC, the indicated detergents or 1000 ng/ml r28M in a final volume of 4 ml. After 24 and 48 hours, respectively, dead tumor cells and PBMC were removed by multiple washing steps with PBS. Remaining cells were lysed by adding hot sodium dodecyl sulfate (SDS) sample buffer (62.5 mM Tris-HCl, 2% SDS, 10% glycerol, 50 mM dithiothreitol). Proteins were separated by SDS-PAGE and transferred to nitrocellulose membranes. After blocking with 5% skim milk powder in TBS/T buffer (25 mM Tris, 0.15 M NaCl, 0.05% Tween® 20, pH 7.5) for one hour at room temperature, the membranes were probed with the primary antibody, either monoclonal rabbit anti-caspase-7 (cat.no. 12827, clone D2Q3L, Cell Signaling; Danvers, USA), monoclonal rabbit anti-cleaved caspase-7 (cat.no. 8438, clone D6H1, Cell Signaling), monoclonal rabbit anti-caspase-3 (cat.no. 9665, clone 8G10, Cell Signaling) or monoclonal rabbit anti-cleaved caspase-3 (cat.no. 9664, clone 5A1E, Cell Signaling) in a 1:1000 dilution in TBS/T buffer containing 5% bovine serum albumin (BSA) over night at 4°C. Goat anti-rabbit-IgG-HRP (1:2000) was used as secondary antibody (cat.no. 7074, Cell Signaling). The proteins were visualized using the ECL detection system (Pierce; Rockford, USA) according to the manufacturer’s instructions.

#### Tryptic digestion and mass spectrometric identification of proteins

The bands of interest were excised from the gel, destained in 0.05 M ammonium carbonate with 48% ethanol and finally dried in acetonitrile. After reduction and alkylation the peptides were eluted and purified using C18 ZipTips (Pierce; Rockford, USA) according to the manufacturer’s protocol. The samples were spotted on the pre-coated target containing α-cyano-4-hydroxycinnamic acid and analyzed by matrix-assisted laser desorption/ionization (MALDI) tandem mass spectrometry (MS/MS) using a Bruker Ultraflex II TOF/TOF device at the VetCORE-Facility for Research (University for Veterinary Medicine; Vienna, Austria), as described previously [29]. Database searches were performed using the NCBI protein database (NCBInr_120131) as well as a customized in-house database containing the r28M peptide spectrum.

#### ELISA

We established a competitive c-myc ELISA to quantify the r28M content in bovine plasma and products after the described purification steps. Briefly, coating was performed with 5 ng/μl of a self-made c-myc-BSA conjugate that was manufactured by mixing the dissolved peptide and protein together and adding a 2% glutaraldehyde solution. The c-myc-peptide (cat.no. M2435, Sigma-Aldrich, St. Louis, USA) served as standard protein. The standard, the samples, as well as the detection antibody (1:2500) (monoclonal mouse anti-myc-HRP, cat.no. R951-25, Invitrogen/Life Technologies) were applied at once. Samples were titrated in triplicates of seven 3-fold serial dilutions.

BSA amounts were also determined by an in-house developed competitive ELISA. Here, the albumin standard from the BCA Protein Quantification Kit (Pierce) was diluted and used for coating purposes and as ELISA standard (starting concentration: 5 μg/ml). Again, the standard, the samples, as well as the detection antibody (1:800 dilution) (sheep anti-BSA-HRP antibody, cat.no. SAL-10P, ICL; Portland, USA) were applied at once. Samples were titrated in triplicates of seven two-fold serial dilutions.

Immunoglobulin G (IgG) quantification was performed by a sandwich ELISA, using a 1:16000 diluted polyclonal rabbit anti-bovine IgG (cat.no. B5645, Sigma-Aldrich) as coating antibody. The bovine gamma globulins standard from the BCA Protein Quantification Kit (Pierce) was diluted (starting concentration: 1 μg/ml) and used as standard protein for the assay. Samples were titrated in triplicates of seven 2-fold serial dilutions, the polyclonal HRP-labeled goat anti-bovine IgG (H+L) diluted 1:2000 (cat.no. 14-12-06, KPL; Gaithersburg, USA) served as detection antibody.

For the analysis of T cell activation and subsequent measurement of interleukin-2 (IL-2), 96 well-plates were coated with mouse anti-human CD3 (cat.no. 16–0039, clone: HIT3a, eBioscience), mouse anti-human CD28 (cat.no. 16–0288, clone: CD28.6, eBioscience; Vienna, Austria), both of them (ratio 1:2), monomeric r28M, and PBS, respectively, in triplets and incubated at 4°C overnight. Each coating antibody was applied at the concentration of 8 μg/ml. After washing with PBS, 1 x 10^5^ PBMC diluted in cell culture medium were added to each well. The IL-2 secretion of these cells was determined after 30 minutes, 2 hours, 8 hours and 24 hours of incubation, respectively. The triplets of each time point were pooled and analyzed using the Human IL-2 (2^nd^ generation) ELISA Ready-SET-Go! Kit (eBioscience) according to the manufacturer’s instructions.

All ELISAs were analyzed by measuring the optical densities at 450 nm using an ELISA reader (Epoch Microplate Spectrophotometer, Biotek; Bad Friedrichshall, Germany). The OD 450 nm values and log r28M concentrations were plotted by using a 4 parameter logistic regression model analysis according to the following equation: F(x) = ((A-D) /(1+ ((x/C) ^B))) + D, where F(x) corresponds to the OD response, x to the used concentration, A to the OD of the blank, B to the hill slope, C to the inflection point and D to the maximum asymptote, the response value for infinite standard concentration.

### Statistical analysis

The target variables (generally optical density readings) were log transformed prior to analyses to avoid dependency of the variance on the mean. Linear models, generally unifactorial or bifactorial ANOVAs with time or excipient as factors, were performed using the program package "R" [30].

## Results

### Optimization of the purification protocol for large-scale application

**Fig 1:**
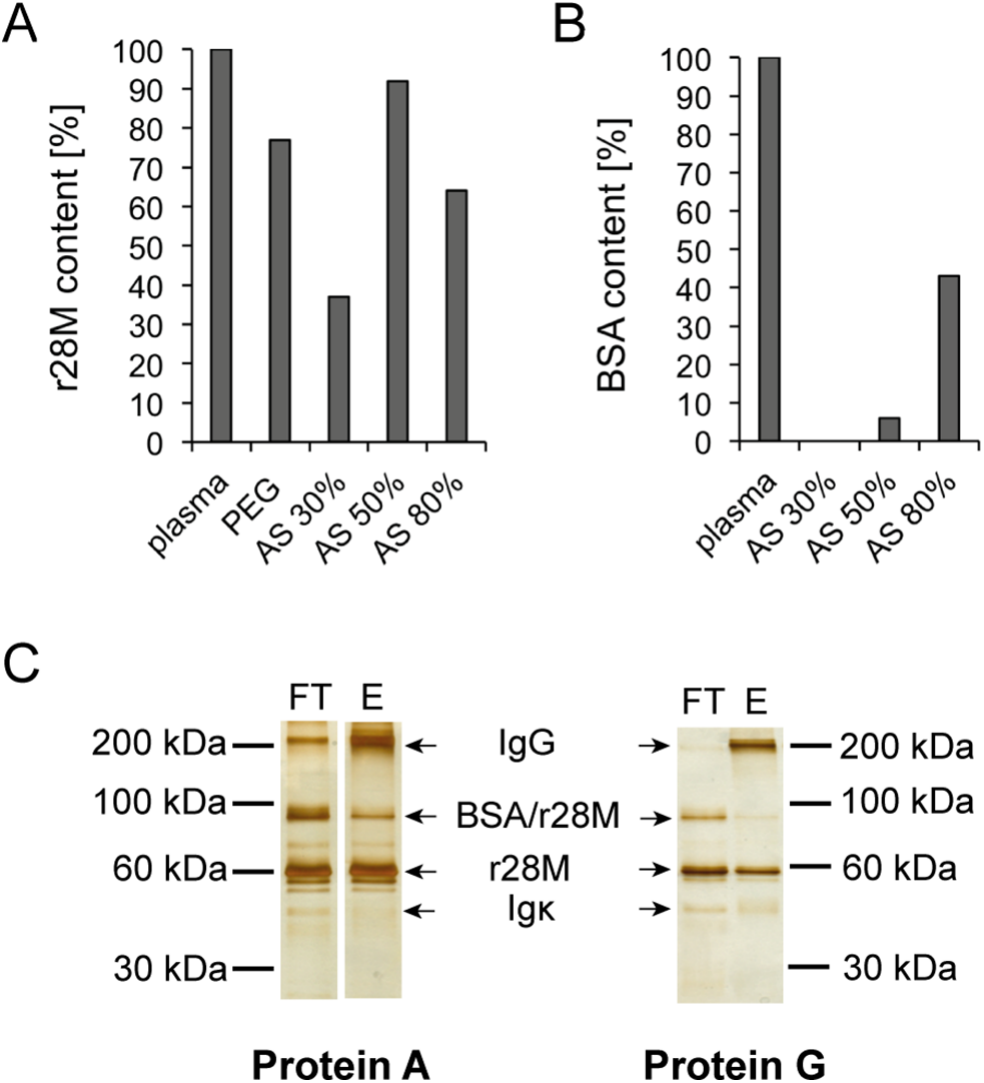
Optimized purification of r28M from plasma from transgenic cattle. The r28M (A) as well as BSA contents (B) of plasma after precipitation with ammonium sulfate (AS) or polyethylene glycol (PEG) were determined by competitive ELISAs and depicted as percentages of the bovine plasma feedstock used for purification of r28M. One representative example out of four independent experiments is shown. (C) The flow-through (FT) and eluate fraction (E) of plasma purified via Protein A or G on two different gels are shown. 500 ng of each fraction were separated by a 8% SDS-PAGE under non-reducing conditions and subsequently silver-stained. Cut FT and E sections of Protein A were on the same gel, but not on nearby slots. One representative example out of three independent experiments is shown.

A purification protocol of the bispecific antibody r28M from bovine plasma has been described previously [12]. This protocol was modified for large-scale purification by the following steps. Firstly, the polyethylene glycol (PEG) precipitation step was replaced by ammonium sulfate (AS) precipitation in respect of better solubility. We tested differently concentrated solutions, namely 30%, 50% and 80% AS. A competitive c-myc ELISA showed that the use of the 50% AS solution resulted in the highest r28M recovery rate with 92% (Fig 1A), additionally, the BSA contamination was rather low (6%) as determined by a competitive BSA-ELISA (Fig 1B). Although using the 30% AS solution resulted in only 0.5% BSA contamination, we decided to precipitate with the 50% AS solution, since the difference in r28M recovery was striking (30% AS: only 37% r28M recovery).

Next, r28M-containing eluate was yielded by Protein L purification as already described [12]. For further purification, the eluate was subsequently passed over a Protein A column and the r28M fraction was collected in the flow trough. This step was included, since in contrast to Protein L [31], Protein A is generally known to primarily bind Fc portions of immunoglobulins [32]. The purified r28M fraction was further separated by SDS-PAGE and visualized by silver staining. Besides the purified antibody other protein bands were detected as well. MALDI MS/MS analysis identified these bands as bovine IgG, BSA-r28M complexes and bovine Igκ (S1A and S1B Fig; S1 Table). In addition, Western Blot analysis revealed the band below the r28M (57 kDa) as BSA protein (S1C Fig). Due to these impurities and the fact that bovine IgG was still detected in the purified r28M fraction, the Protein A column was substituted by a Protein G column. Protein G is reported to bind heavy chains and in contrast to Protein A shows an affinity for all isoforms of IgG and albumin [31]. This step clearly improved the removal of IgG, as shown by sections of a silver-stained SDS-PAGE (Fig 1C). The protein amounts and corresponding IgG contents, as determined by a BCA assay and an IgG ELISA, respectively, showed a removal of 85% IgG, when using Protein A, compared to 94% when Protein G was used. In addition, the purification via a Protein G column also led to decreased amounts of the former identified BSA-r28M complexes (Fig 1C). Analysis using the Quantity One Software (Version 4.6.9) revealed a reduction of 12% when using Protein G (Protein A: 36% BSA/r28M complexes; Protein G: 24% BSA/r28M complexes).

With this optimized protocol we obtained an average of 30 mg enriched purified r28M fraction out of 2 liters bovine plasma (n = 4, SE = 2.59) in a single purification step as determined by BCA assay.

### Specificity and time dependence of r28M activity

**Fig 2:**
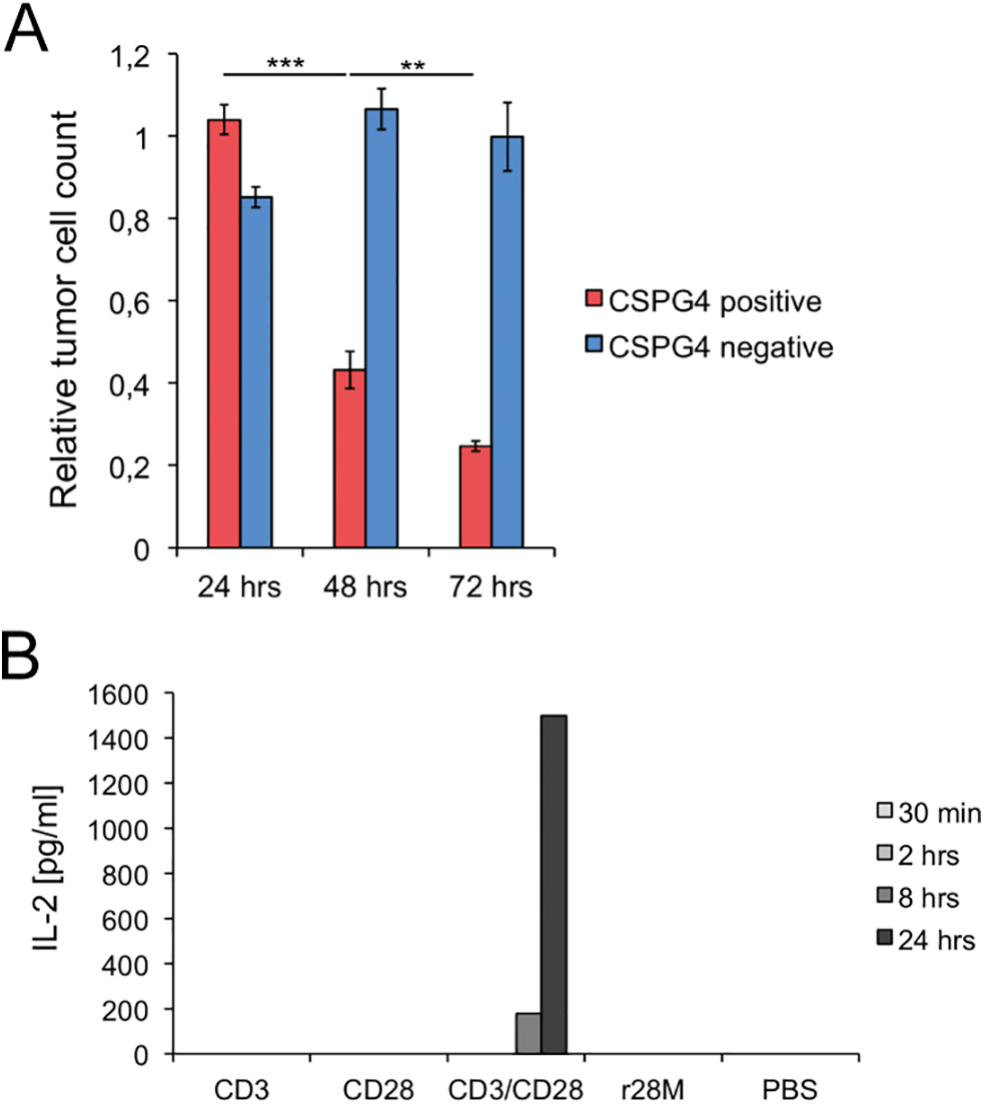
Specificity and time dependence of the biological activity of r28M. (A) CSPG4 positive (IPC-298) or negative cells (U-251 MG) were incubated together with PBMC and 1000 ng/ml enriched r28M fraction in a final volume of 150 μl for 24, 48 as well as 72 hrs to measure tumor cell viability. Results show samples normalized to the controls. Cells incubated without r28M served as controls and their number was considered as 1. Values are means ± SE. Significance levels: x: p ≤ 0.05; xx: p ≤ 0.01; xxx: p ≤ 0.001. (B) Plates were coated with the indicated antibodies or PBS and incubated with PBMC for 30 min, 2 hrs, 8 hrs as well as 24 hrs, respectively. Preparations were pipetted in triplicates. The supernatants of these triplicates were pooled and the IL-2 concentrations were measured by ELISA.

Subsequently, the activation and specificity of purified r28M was tested. The temporal peak activity of the purified enriched r28M fraction was analyzed after 24, 48 and 72 hours of co-incubation with tumor cells and PBMC (Fig 2A). No significant effect on tumor cell viability was observed after 24 hours. After 48 hours 43% of CSPG4 positive tumor cells were still alive, whereas only 25% viable tumor cells were left after incubation for 72 hours. The viability of the CSPG4 negative tumor cells was not affected by the addition of r28M at any time (Fig 2A).

To confirm the specific activity of the antibody, or to be more precise, the tumor cell dependence of T cell activation by the r28M antibody, the IL-2 secretion by PBMC as a crucial factor in T cell activation [33] was investigated. As shown in Fig 2B, only the combined application of CD3 and CD28 led to IL-2 secretion already after 8 hours (< 200 pg/ml) and an increase up to 1500 pg/ml after the incubation for 24 hours. In contrast, r28M as well as CD3 and CD28 alone, did not lead to IL-2 secretion and thus to no activation of PBMC in the absence of tumor cells (Fig 2B).

### Detergents enhance the biological activity of r28M

In consideration of future applications of the bispecific antibody r28M, the effect of the addition of different possible excipients (Tween® 20, Tween® 80, sodium-deoxycholate (Na-deoxycholate) and saponin) was evaluated. First, to exclude any effect resulting from the detergents per se, the viability of PBMC and/or CSPG4 positive cells in the presence of different concentrations were analyzed by measurements of the optical density (S2A and S2B Fig). The used detergents and concentrations are given in S2C Fig.

The following concentrations of detergents did not influence cell growth of neither PBMC nor CSPG4 positive tumor cells positively or negatively: Tween® 20 (0.0067%, 0.0013%), Tween® 80 (0.0067%, 0.0013%), saponin (0.0067%), and Na-deoxycholate (0.0013%).

**Fig 3:**
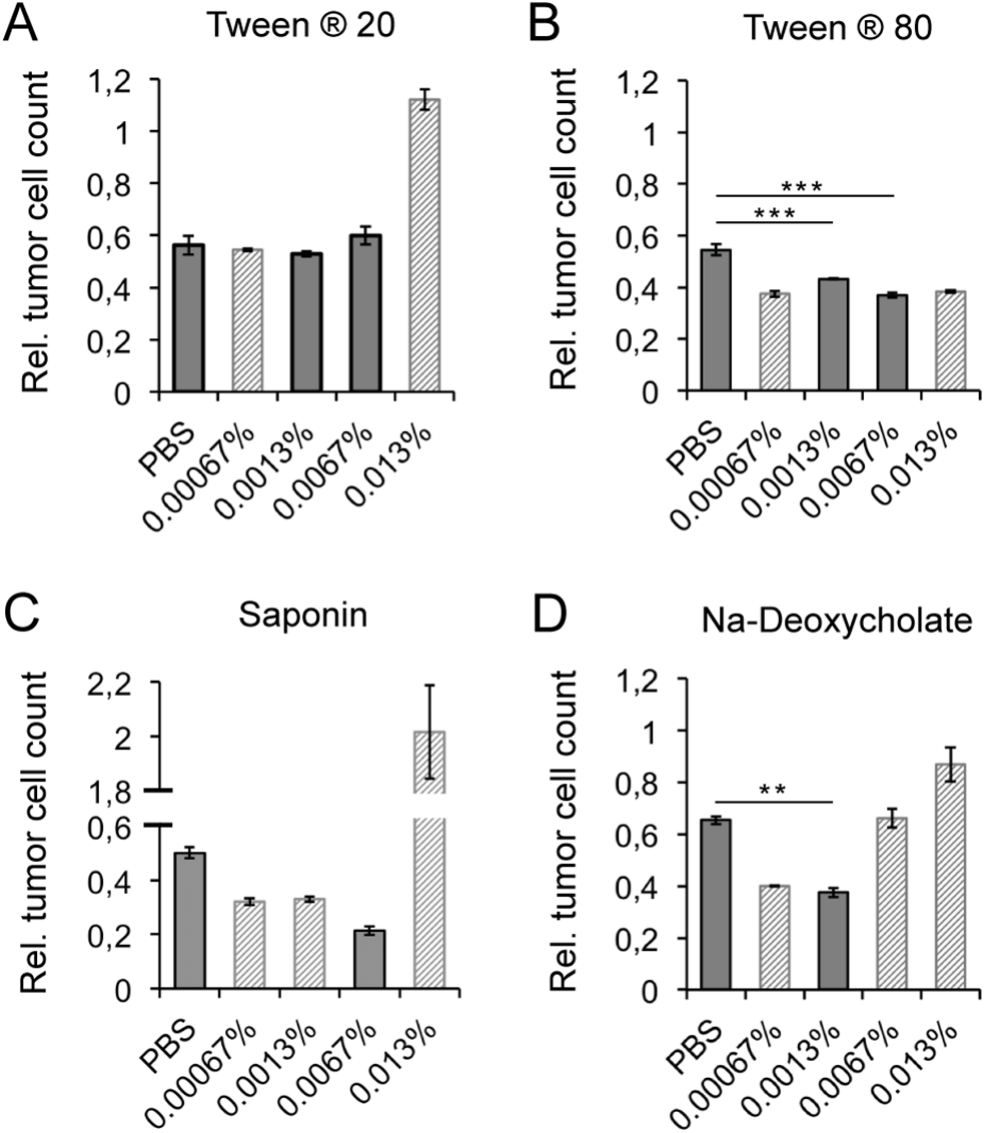
Impact of detergents on the r28M activity. The effects of the addition of the detergents Tween® 20 (A), Tween® 80 (B), saponin (C) and Na-deoxycholate (D) on the efficacy of PBMC to kill CSPG4 positive tumor cells (IPC-298) induced by 1000 ng/ml enriched r28M fraction are shown. Full grey bars: considered concentration of detergents (no positive or negative influence on the growth rate of CSPG4 positive cells and/or PBMC—see S2 Fig). Dashed bars: concentration of detergents that influenced cell growth either positively or negatively (see S2 Fig); therefore data was not included in the given statistical analysis. Results show samples normalized to the controls. Cells incubated without r28M served as controls, their number was considered as 1. Values are means ± SE. Significance levels: x: p ≤ 0.05; xx: p ≤ 0.01; xxx: p ≤ 0.001.

1000 ng/ml of the bispecific antibody r28M were then charged with detergents in evaluated concentrations and analyzed by a cell viability measurement assay (Fig 3A–3D). The addition of 0.0067% and 0.0013% Tween® 80, as well as the addition of 0.0013% Na-deoxycholate, had beneficial effects on the biological activity of r28M. The killing of CSPG4 positive tumor cells was enhanced by 11% and 17% (0.0067% and 0.0013% Tween® 80) and 28% (0.0013% Na-deoxycholate), respectively, without interfering with cell growth (Fig 3B and 3D).

Those detergent concentrations, which had a significant impact on the viability and growth of PBMC and/or CSPG4 positive cells, either in a positive or negative way, were tested and included in Fig 3, but omitted from statistical analysis and not considered for further application.

### Secondary purification steps exposed the monomer as being the most appropriate r28M molecule for further applications

**Fig 4:**
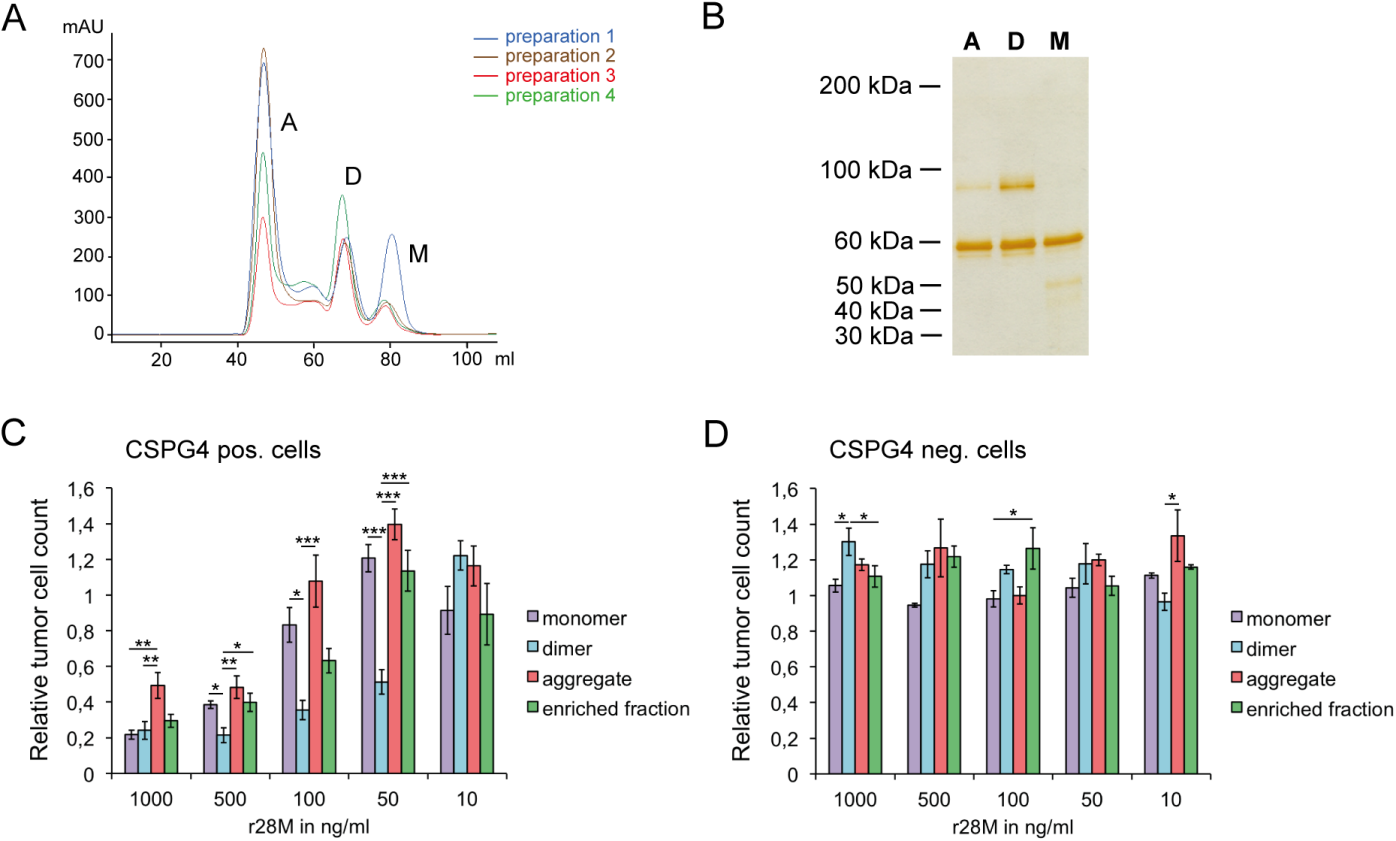
Analysis of the different r28M fractions after size exclusion chromatography (SEC). (A) The chromatograms of 4 independent purification batches after separation via SEC show the monomer (M), dimer (D) and aggregate (A) fractions. (B) Silver staining of the fractions (500 ng per lane) separated by SDS-PAGE under non-reducing conditions. A = aggregate, D = dimer, M = monomer. Results are shown from one representative out of 10 independent experiments. **(**C and D) CSPG4 positive cells (IPC-298) (C) as well as CSPG4 negative cells (U-251 MG) (D) were incubated together with PBMC and 10–1000 ng/ml of the different r28M fractions in a final volume of 150 μl/well. After 72 hrs the amount of viable tumor cells was determined. Results show samples normalized to the controls. Cells incubated without r28M served as controls, their number was considered as 1. Values are means ± SE. Significance levels: x: p ≤ 0.05; xx: p ≤ 0.01; xxx: p ≤ 0.001.

Bispecific antibodies occur in different configurations and form dimers and/or aggregates under certain conditions [34]. For this reason and to enhance purity, we separated the purified enriched r28M fraction by SEC (Fig 4A). Three different r28M formations, namely monomers, dimers and aggregates, were detected, separated by SDS-PAGE and visualized by subsequent silver staining (Fig 4B). Although the impurities could be reduced markedly, all preparations still showed additional protein bands: the r28M monomer showed a band of about 50 kDa, the 90 kDa protein band was observed in the dimer and as well but to a lesser extent in the aggregate fraction. Measurements of the BSA content by a competitive sandwich ELISA revealed a BSA contamination of 4.95% in the dimer fraction and only 0.34% in the monomer fraction.

Cell viability measurements revealed that the r28M dimer generally exhibited the highest efficiency in inducing PBMC to kill CSPG4 positive tumor cells (Fig 4C). Differences were observed between the activity of monomers and dimers at concentrations of 50–500 ng/ml, with dimers inducing significantly more tumor cell killing. The application of 1000 ng/ml as well as 10 ng/ml r28M revealed no differences in actions between monomers and dimers. There were also no differences between the activity of monomers and the enriched r28M fraction and the aggregates, respectively, at all tested concentrations, except the use of 1000 ng/ml r28M aggregate, which resulted in a higher tumor cell viability than the application of 1000 ng/ml of the other r28M fractions. Furthermore, the application of some fractions led to enhanced growth of CSPG4 positive and negative cells, but CSPG4 negative tumor cells could not be killed with any of the fractions at any concentration (Fig 4D).

The aggregate fraction showed the least impurities, but protein aggregation may have a negative impact on protein functions [35]. Hence we decided to exclusively follow up with the r28M monomer, since its content is defined, the amount of impurities is minor and there were no differences in the biological activity between monomer and dimer at r28M concentrations exceeding 500 ng/ml. Moreover, the 50 kDa bovine Igκ, detected in the monomer fraction, does not seem to have influence on the viability of tumor cells, as tested on CSPG4 negative cells, where no differences between tumor cells incubated with PBMC together with monomeric r28M or without it could be observed (Fig 4D).

### A single 1000 ng/ml dose of r28M is sufficient to enfold its full biological activity

**Fig 5:**
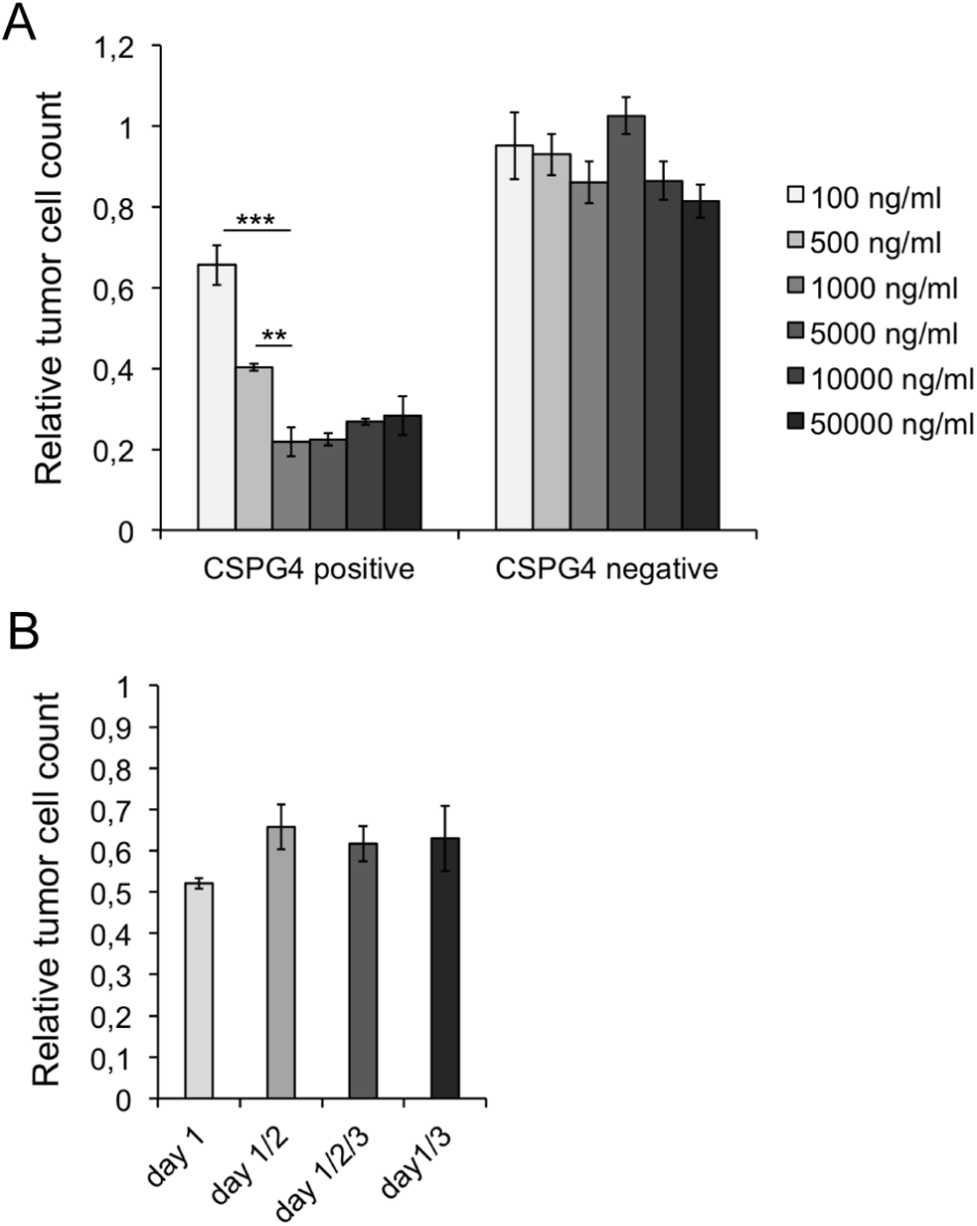
Dose and time dependency of r28M actions. Results from tumor cell viability measurements show samples normalized to the controls. Cells incubated without r28M served as controls, their number was considered as 1. Values are means ± SE. Significance levels: x: p ≤ 0.05; xx: p ≤ 0.01; xxx: p ≤ 0.001. PBMC from two different donors were used for the shown experiments. (A) CSPG4 positive (IPC-298) and negative cells (U-251 MG) were incubated with PBMC and monomeric r28M in concentrations ranging from 100–50000 ng/ml for 72 hrs in a final volume of 150 μl. (B) CSPG4 positive cells (A-375) were incubated with PBMC and 1000 ng/ml monomeric r28M for 72 hrs. 1000 ng/ml r28M were applied either immediately (day 1), immediately and with a repeated administration after 24 hrs (day 1/2), immediately and after 24 and 48 hrs (day 1/2/3) or immediately and after 48 hrs (day 1/3).

To determine the r28M concentration that is sufficient to bind 10^5^ PBMC as well as 10^4^ CSPG4 positive tumor cells, 100 up to 50000 ng/ml monomeric r28M were applied in a cell viability measurement assay in a final volume of 150 μl. The maximum killing efficiency, as determined by the measurement of viable tumor cells, was reached with a concentration of 1000 ng/ml, in our experimental set-up corresponding to 150 ng. An increase of r28M dose had no impact on the efficiency of killing CSPG4 positive cells. CSPG4 negative cells were not affected by any applied concentration of r28M (Fig 5A).

Furthermore the effects of repeated applications were examined. 1000 ng/ml monomeric r28M were applied in the context of a cell viability measurement either once (day 1), twice (day 1 and 2 or day 1 and 3) or three times (day 1, 2 and 3). There were no significant differences in tumor cell viability between these treatments, indicating that a single dose of 1000 ng/ml monomeric r28M is sufficient for displaying its full biological activity in our experimental set-up (Fig 5B).

### r28M enhances a caspase 3 and 7 related apoptotic pathway

**Fig 6:**
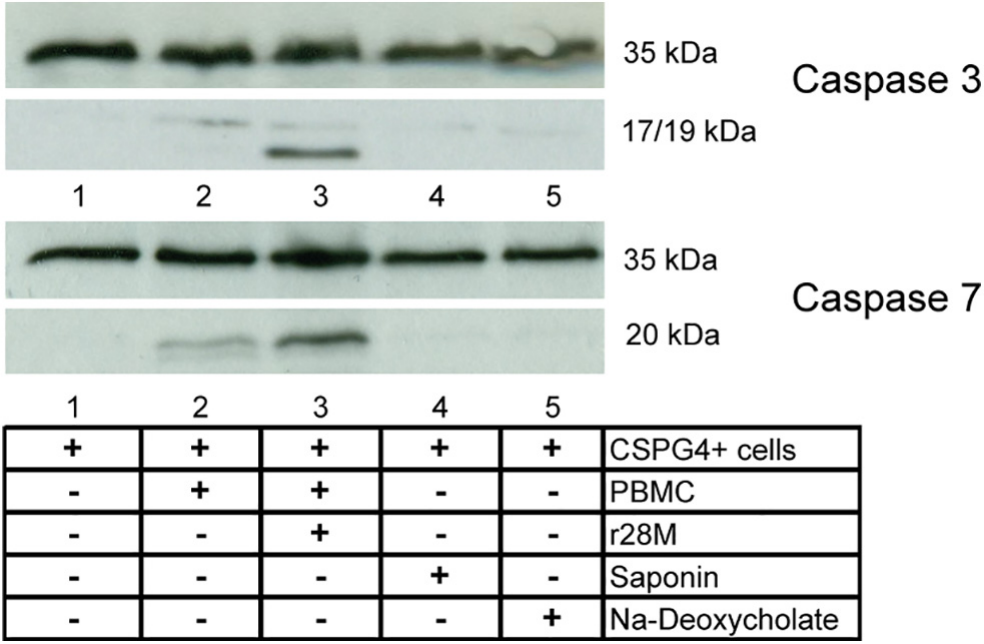
Caspase 3 and 7 release of cells incubated with r28M or detergents. CSPG4 positive cells (IPC-298) were incubated for 24 hrs as indicated (saponin and Na-deoxycholate: [c] = 0.0067%; 1000 ng/ml r28M). Cells were lysed, cell proteins were separated by SDS-PAGE and subsequently investigated by a Western Blot with an anti-caspase 3 (35 kDa), anti-cleaved caspase 3 (17 and 19 kDa), anti-caspase 7 (35 kDa) or anti-cleaved caspase 7 (20 kDa) antibody. One representative out of 4 independent experiments is shown each.

To gain a deeper insight into the cellular pathways that might be induced by r28M-activated PBMC, we investigated the activation of caspase 3 and 7 in tumor cells. Therefore, we examined tumor cells that had been incubated with PBMC either together or without r28M by Western Blot analysis using specific antibodies against caspase 3 and 7. Tumor cells incubated with harmful concentrations of saponin and Na-deoxycholate were also analyzed. First of all, we did not observe any differences in caspase protein levels after 24 and 48 hours of incubation of cells with detergents or r28M. The inactive 35 kDa pro-caspases were ubiquitously expressed by all tumor cells at all tested conditions (Fig 6). CSPG4 positive tumor cells, incubated together with PBMC and r28M, underwent a caspase 3 and 7 related pathway, strong signals of the caspase 3 subunits (17 and 19 kDa) and caspase 7 subunit (20 kDa) were detected (Fig 6, lane 3). Similar results were obtained for CSPG4 positive cells incubated with PBMC but without r28M. Here, the subunit signals were weaker but still detectable. No caspase subunits were observed when CSPG4 positive cells were incubated with differing harmful concentrations of the tested detergent but without r28M (Fig 6). Generally, the differentiation between apoptosis and necrosis is questionable at different cell stages, but here we could clearly highlight the activation of tumor cell caspases by r28M-activated PBMC.

### T cells are mainly responsible for the direct lymphocyte answer to an r28M administration

**Fig 7:**
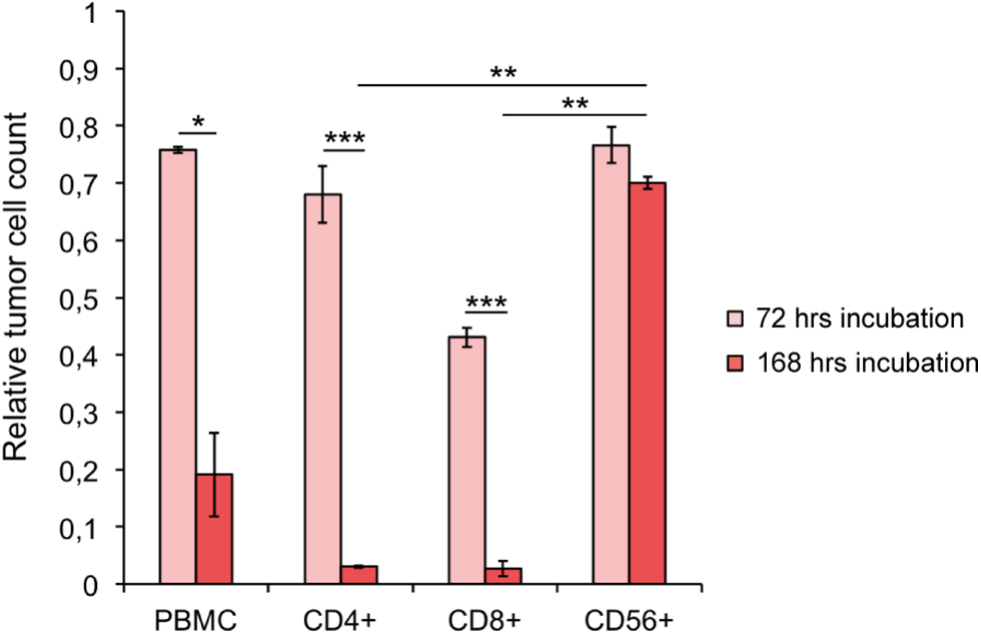
Killing efficiency of lymphocyte subpopulations. CSPG4 positive cells (A-375) were incubated with PBMC, CD4^+^, CD8^+^ and a CD56^+^ enriched cell fraction, respectively, together with 1000 ng/ml monomeric r28M in a final volume of 150 μl. The amount of viable tumor cells was determined after 72 and 168 hrs of incubation. Results show samples normalized to the controls. Cells incubated without r28M served as controls, their number was considered as 1. Values are means ± SE. Significance levels: x: p ≤ 0.05; xx: p ≤ 0.01; xxx: p ≤ 0.001.

To dissect the cell types involved in response to r28M, freshly isolated PBMC were separated via MACS® technology into CD4^+^ helper T cells, CD8^+^ cytotoxic T cells and CD56^+^ NK and NK-T cells. Subsequently, the cell population homogeneity was analyzed via FACS and an average purity of 99% for CD3^+^CD4^+^ and 88% for CD3^+^CD8^+^ cells could be achieved. Although the order of selection for isolating CD56^+^ cells via MACS® technology was tested in different ways, an enriched fraction containing a maximum of 62% CD56^+^ cells and 14% CD3^+^CD56^+^ NKT cells could be achieved. However, CD56^+^ cells always displayed the prominent fraction. Unsorted PBMC and the above mentioned three separate cell populations, respectively, were applied in a cell viability measurement assay. The effects, caused by these lymphocytes activated by 1000 ng/ml monomeric r28M, on tumor cells have been investigated after 72 hours and 168 hours of incubation in a final volume of 150 μl. After 72 hours moderate CSPG4 positive cell killing (23–57%) and no significant differences in the killing efficiency of all applied lymphocyte fractions were observed (Fig 7). After 168 hours the percentage of CSPG4 positive tumor cells killed by the CD4^+^ and CD8^+^ cell fraction rose up to 97%, as only 3% viable tumor cells were left. Unsorted PBMC killed 81% CSPG4 positive tumor cells, here 19% viable cells were left. There was no difference in CD56^+^ mediated cell action between 72 hours (77% viable tumor cells left) and 168 hours (70% tumor cells left) of incubation, suggesting that CD56^+^ cells play only a minor role in direct tumor cell killing actions induced by r28M.

## Discussion

More than 20 years ago, the first transgenic farm animal was cloned from nuclear transfer using adult somatic donor cells [36]. Since then major progress has been made in the field of transgenesis and reproductive technologies, allowing the generation of genetically modified large animals for the production of human health products, including monoclonal and polyclonal antibodies. Transgenic animals have considerable advantages over bacterial or yeast-systems. Beside the appropriate mammal-specific expression and folding, proteins undergo post-translational modifications and glycosylation. This is also valid for mammalian cell culture systems, but compared to transgenic animals, production levels are low. Furthermore the up-scaling of cell culture systems is a major cost-intense and time-consuming problem [26].

Although, compared to pigs, sheep and goats, cattle have the longest gestation time, highest age at sexual maturity and fewest offspring, no other animal produces such high protein amounts constantly over a long period of time. If a protein is expressed in bovine milk, an annual yield of 40 kg protein can be achieved, about 10 times more than in caprine milk, as reviewed by Houdebine [26]. The need for perfectly sterile conditions when proteins are collected for the use as drugs emphasizes bovine blood as a production site for therapeutic antibodies, since the blood circuit is a closed production system that prevents contamination with human pathogens if certain GMP requirements are met.

Within 3 years we built up a GMP-compliant production unit, including a housing, feeding and collection system (approved by the government of Upper Bavaria for Paktis Antibody Services GmbH; permission number: DE_BY_04_MIA_2011_0196/53.2-ZAB-2671.1 P220). We managed to generate r28M-transgenic animals with good health status, as checked regularly by our in-house veterinary. The r28M-expression of these animals increased with age, as the adult animals express 130–560 μg/ml r28M in their blood. About 4 liters plasma can be gained from cattle per day, resulting in 60 mg purified r28M per plasmapheresis. Since the average life expectancy of cattle is about 20 years, a stable and closed production system would be available once the transgenic animal is generated.

Up to now, to our knowledge no data on the production or purification of a bispecific scFv antibody from blood of a transgenic farm animal are available. Thus, this study is the first to deal with the large-scale production of a bispecific antibody, namely r28M, in transgenic cattle.

Initially, the antibody was purified via a PEG precipitation followed by purification by Protein L and gel filtration. This resulted in a protein fraction consisting of 50–70% r28M [19]. In this study we focused on the large-scale purification of the r28M antibody from bovine plasma and included several optimization steps to yield more antibody with reduced impurities. To achieve these objectives, we substituted the initial PEG precipitation by precipitation via ammonium sulfate and included an additional purification step via Protein A. This was later exchanged to purification via Protein G, which reduced the contaminants from bovine plasma markedly. Next, the enriched r28M fraction was separated by SEC into monomers, dimers, and aggregates. Due to non-specific binding of proteins to the column [37], the r28M content was lower, but also the impurities could be minimized. Aggregation is driven by contamination [37], which besides non-protein material also includes damaged forms of the r28M. Thus, working with a purified r28M monomer, which only included minor Igκ impurities but no r28M-BSA complexes is preferable. We tried to optimize the separation of Igκ from the r28M monomer by testing different solvents. Using a low salt buffer (S3 Fig) we could obtain a higher resolution and the single r28M monomer peak separated into two, which might facilitate a more stringent purification. Nevertheless, the signal intensity was remarkably lower and also a shift of retention time was observed when using the low salt buffer.

In the present study we could verify that not only cytotoxic CD8^+^ T cells are involved in r28M-mediated tumor cell killing but also CD4^+^ cells. This has also been previously reported for a superagonistic anti-CD28 antibody [38]. Further, Grosse Hovest et al. already described that non-T cells significantly contribute to the lytic activity of r28M-activated PBMC towards CSPG4^+^ cells but also stated that they are not active if the isolated fractions are used in a long-term tumor viability measurement assay [12, 19]. Our results confirm this assumption. The used CD56^+^ enriched cell population is composed of only 43–62% CD56^+^ cells and an average of 30% CD8^+^ cells. The CD56^+^ enriched cell population killed only 30% of CSPG4 positive cells within 168 hours, whereas the incubation with every other cell fraction for 168 hours resulted in more than 80% killed CSPG4^+^ cells (Fig 7). Thus, we conclude that CD56+ cells might only play a minor role in r28M-mediated tumor cell killing in our experimental set-up. This is also supported by the fact that the r28M has no Fc-portion to deliver cell-mediated cytotoxicity. However, the high IL-2 concentration secreted by activated PBMC as well as more than 5000 pg/ml of other cytokines like interferon-gamma, IL-5 and tumor necrosis factor alpha and beta (data not shown) is known to lead to the activation and differentiation of immune cells other than T cells. But with respect to r28M, these complex mechanisms still need to be investigated. We note that these cytokines were exclusively secreted, if CSPG4^+^ cells were co-incubated with lymphocytes and the r28M antibody.

The use of frozen PBMC from different donors contribute to the variation observed in the individual tumor cell viability measurement experiments.

Our observation that the apoptotic pathway of CSPG4 positive tumor cells is activated by r28M-mediated PBMC is in accordance with Bäuerle and Stein, who state that connecting a tumor cell with an effector cell through an immunologic synapse mediated by a bispecific antibody causes cytolysis of the tumor cell [16, 39]. They suppose that other apoptosis, necrosis or autophagy signals may be sent into the target cell as a consequence of the multiple interactions between target and effector cell in the synapse [16]. Mature caspase 3 and 7 are known to be the major downstream executioners of the apoptotic process. Denecker et al. showed that cleaved forms of caspase 3 and 7 are released in the cell culture supernatant if apoptosis was induced. Otherwise, in the case of necrosis no subunits can be detected [40]. This is in line with our results, indicating apoptotic processes induced by r28M, whereas the addition of harmful concentrations of the surfactants saponin and Na-deoxycholate resulted in the activation of necrosis, as evidenced by Western Blot analysis using antibodies against caspase 3 and 7 subunits. Although the differentiation between apoptosis and necrosis is difficult at certain cell death stages [41], we could demonstrate a clear difference between the r28M-induced effect of PBMC and the impact of a detergent that is toxic to the tested cells at the used concentrations.

Proteins are only marginally stable and prone to physical degradation. Thus, an excipient was added to the r28M to enhance its stability by suppressing aggregation, the primary pathway of protein physical degradation, surface adsorption or by maintaining physiological osmolarity [42]. The positive effects of excipients have often been described [43] and the European Medicines Agency (EMA) provides guidance on excipients for application for marketing authorization of medicinal products. We chose four different detergents in low concentrations that should protect the antibody from surface and stress induced aggregation [44]. Since two out of four tested detergents resulted in beneficial effects on the antibody’s efficiency, future work on r28M will include the testing of more detergents regarding their influence on the biological activity of r28M.

These days, there are multiple ways to fight cancer and progress has been made in the resection of solid tumors, but the targeting of metastases is still a big challenge. Due to the biological heterogeneity of metastatic cells and difficulties to detect them, metastasis is the most deadly feature of cancer, causing more than 90% of cancer-related mortality [45, 46]. Bispecific scFv antibodies as the r28M represent a promising tool for directly targeting metastatic cancer cells by T cell mediated cytotoxicity.

## Supporting Information

S1 Fig Investigation of undesired proteins after r28M purification. (A) The enriched r28M fraction purified via Protein A was separated by SDS-PAGE under non-reducing conditions and subsequently silver stained. (B) The undesired proteins, namely all proteins except r28M (57 kDa) were cut out, and analyzed by SDS-PAGE under non-reducing (bands X and Y) as well as reducing conditions (bands X1, X2, Y1) and subsequent silver staining. Accurate identification of these proteins was performed by mass spectrometry and is shown in S1 Table. (C) Anti-BSA and anti-c-myc (detection of r28M) Western Blots of the non-reduced enriched r28M fraction, purified via Protein A, are shown.
(TIF)

S2 Fig Effect of different detergents on cell viability. PBMC (A) or CSPG4 positive cells (IPC-298) (B) were incubated together with the depicted concentrations of either Tween® 20, Tween® 80, saponin or Na-Deoxycholate (Na-deoxy). Cell viability was determined after 72 hrs of incubation and is represented as optical density (OD) at 450 nm. Values are means ± SE. Significance levels: x: p ≤ 0.05; xx: p ≤ 0.01; xxx: p ≤ 0.001. (C) Detergent concentrations which did not influence cell growth of neither PBMC nor CSPG4 positive tumor cells (IPC-298) were chosen for further analyses and are highlighted in yellow. Significance levels are given for concentrations influencing cell growth.
(TIF)

S3 Fig Effect of different buffers on the SEC profile of r28M. The enriched r28M fraction was separated by SEC using PBS, high salt buffer (HSB) or low salt buffer (LSB). The corresponding profiles are depicted as follows: A = aggregate, D = dimer, M = monomer.
(TIF)

S1 Table Mass spectrometric based identification (data affiliating to S1 Fig). (DOCX)
