# Supplementary figures and images for: Large-Scale Purification of r28M: A Bispecific scFv Antibody Targeting Human Melanoma Produced in Transgenic Cattle
Source: PLoS One. 2015 Oct 15;10(10):e0140471. doi: 10.1371/journal.pone.0140471 (PMC4607477; doi:10.1371/journal.pone.0140471)

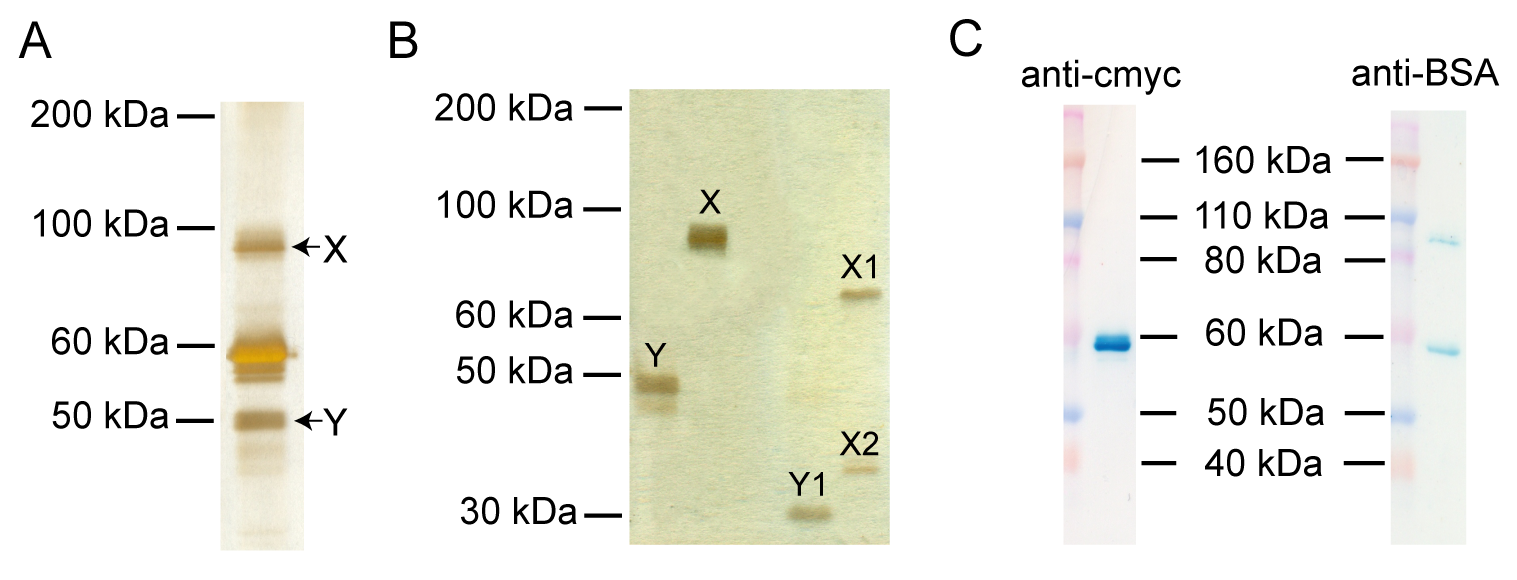

Supplement: S1 Fig — (A) The enriched r28M fraction purified via Protein A was separated by SDS-PAGE under non-reducing conditions and subsequently silver stained. (B) The undesired proteins, namely all proteins except r28M (57 kDa) were cut out, and analyzed by SDS-PAGE under non-reducing (bands X and Y) as well as reducing conditions (bands X1, X2, Y1) and subsequent silver staining. Accurate identification of these proteins was performed by mass spectrometry and is shown in S1 Table. (C) Anti-BSA and anti-c-myc (detection of r28M) Western Blots of the non-reduced enriched r28M fraction, purified via Protein A, are shown. (TIF) [file pone.0140471.s001.tif]

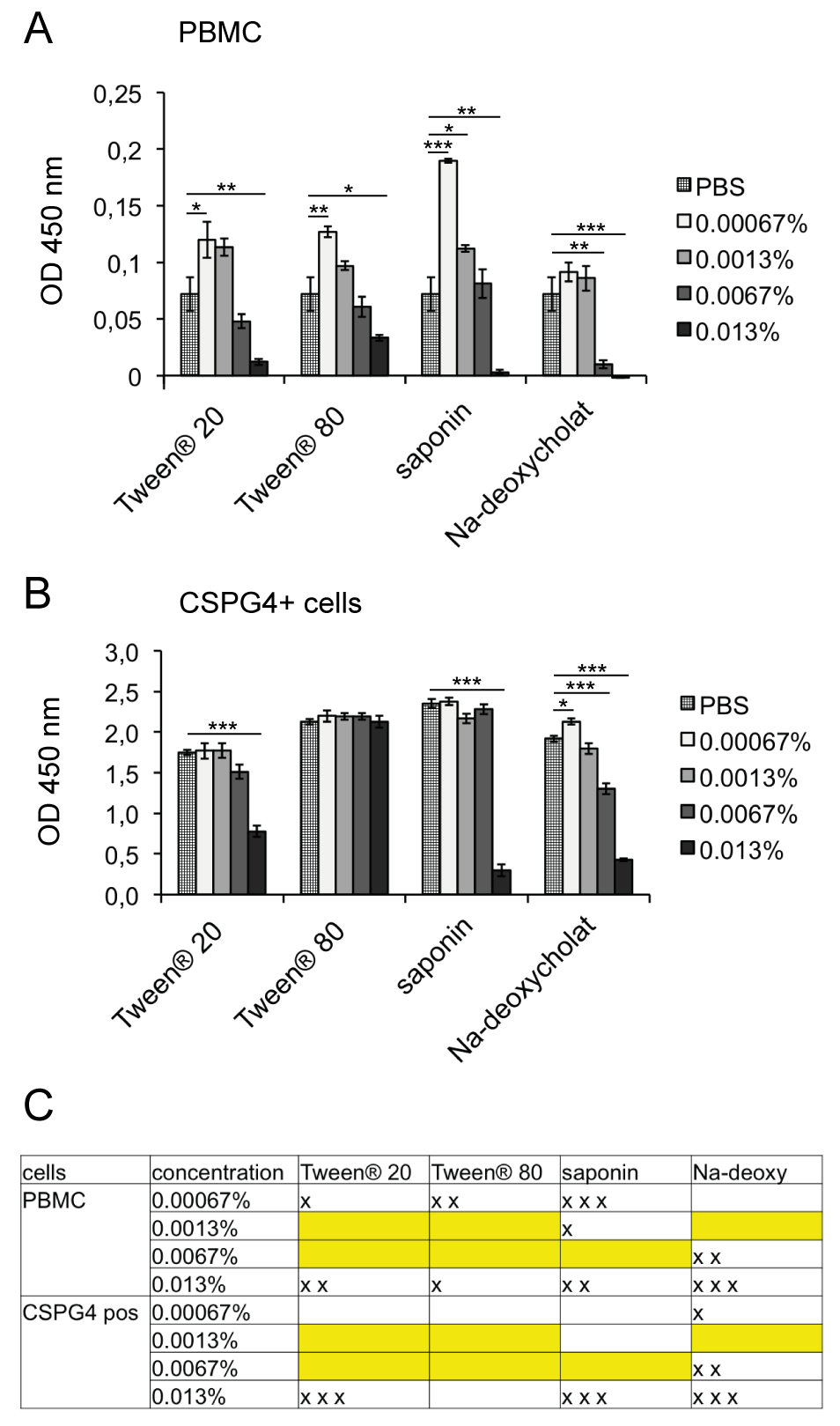

Supplement: S2 Fig — PBMC (A) or CSPG4 positive cells (IPC-298) (B) were incubated together with the depicted concentrations of either Tween® 20, Tween® 80, saponin or Na-Deoxycholate (Na-deoxy). Cell viability was determined after 72 hrs of incubation and is represented as optical density (OD) at 450 nm. Values are means ± SE. Significance levels: x: p ≤ 0.05; xx: p ≤ 0.01; xxx: p ≤ 0.001. (C) Detergent concentrations which did not influence cell growth of neither PBMC nor CSPG4 positive tumor cells (IPC-298) were chosen for further analyses and are highlighted in yellow. Significance levels are given for concentrations influencing cell growth. (TIF) [file pone.0140471.s002.tif]

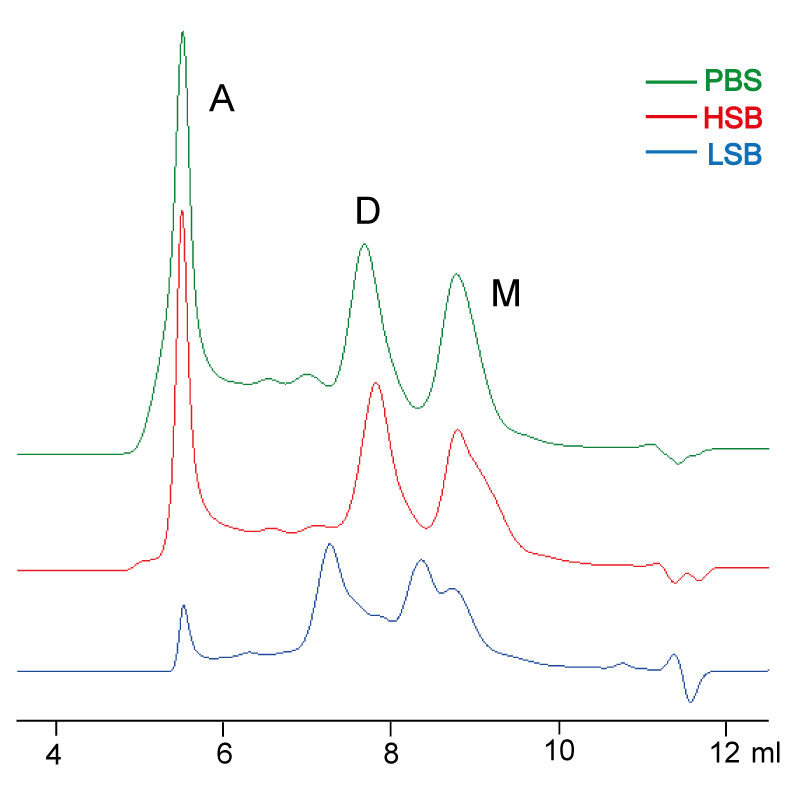

Supplement: S3 Fig — The enriched r28M fraction was separated by SEC using PBS, high salt buffer (HSB) or low salt buffer (LSB). The corresponding profiles are depicted as follows: A = aggregate, D = dimer, M = monomer. (TIF) [file pone.0140471.s003.tif]
