# Supplementary material for: Large-Scale Purification of r28M: A Bispecific scFv Antibody Targeting Human Melanoma Produced in Transgenic Cattle
Source: PLoS One. 2015 Oct 15;10(10):e0140471. doi: 10.1371/journal.pone.0140471 (PMC4607477; doi:10.1371/journal.pone.0140471)
Supplement: S1 Table — (DOCX) [file pone.0140471.s004.docx]

**Supporting Table 1. Mass spectrometric based identification (data affiliating to S1 Fig.).**

| **band** | **accession** | **MW [kDa]** | **score** | **#peptide** | **SC [%]** | **protein** |
| --- | --- | --- | --- | --- | --- | --- |
| **X** | r28M_1_544 | 57.3 | 202.0 | 3 | 8.5 | r28M whole sequence 1-544 |
|  | gi/110561 | 10.3 | 181.3 | 4 | 35.8 | Ig kappa chain V region - mouse |
|  | gi/30794280 | 69.3 | 184.9 | 2 | 4.3 | albumin (*Bos taurus*) |
| **X1** | gi/30794280 | 69.3 | 107.0 | 13 | 7.7 | albumin (*Bos taurus*) |
| **X2** | r28M_1_544 | 57.3 | 363.6 | 4 | 12.9 | r28M whole sequence 1-544 |
|  | gi/110561 | 10.3 | 207.5 | 4 | 35.8 | iG kappa chain V region - mouse |
| **Y** | gi/154425814 | 26.3 | 140.6 | 2 | 14.6 | IGK protein (*Bos taurus*) |
| **Y1** | gi/154425814 | 26.3 | 140.6 | 2 | 14.6 | IGK protein (*Bos taurus*) |

MW = molecular weight; SC = sequence coverage
